# Expression of caspase-3, p53 and Bcl-2 in generalized aggressive periodontitis

**DOI:** 10.1186/1746-160X-2-17

**Published:** 2006-06-20

**Authors:** Şule Bulut, Hilal Uslu, B Handan Özdemir, Ömer Engin Bulut

**Affiliations:** 1 Baskent University, Faculty of Dentistry, Department of Periodontology, Ankara, Turkey; 2 Baskent University, Faculty of Medicine, Department of Pathology Ankara, Turkey; 3 Baskent University, Faculty of Dentistry, Department of Oral and Maxillofacial Surgery, Ankara, Turkey

## Abstract

**Background:**

Apoptosis, or programmed cell death is a form of physiological cell death. It is increased or decreased in the presence of infection, inflammation or tissue remodelling. Previous studies suggest that apoptosis is involved in the pathogenesis of inflammatory periodontal disease. The aim of the present study was to investigate the clinical features and known indicators of apoptosis (p53, Bcl-2, Caspase-3) in patients with generalized aggressive periodontitis (GAP)

**Methods:**

Eight patients with GAP, who had sites with probing depths (PD) > 5 mm, and 10 periodontally-healthy persons were included in the study. Clinical examinations and PD were performed, and the plaque index and gingival index were recorded. Gingival tissues biopsies were obtained from active site of each patient and from healthy individuals. The expression of caspase-3, Bcl-2, and p53 was evaluated by immunohistochemistry

**Results:**

There were no significant differences between GAP and control group with respect to levels of caspase-3 and p53 expression (P > 0.05). Contrary, the frequency of grade 3 expression of Bcl-2 was higher in GAP group than the control group.

**Conclusion:**

The higher frequency of *Bcl-2 *expression in GAP group indicates and delayed apoptosis can lead to increasing resident inflammatory cells in periodontal tissues and resulting in progressive periodontal destruction.

## Background

Inflammatory periodontal diseases are characterized as local and peripheral infection involving multiple species of gram-negative organisms. *Actinobacillus actinomycetemcomitans (aa) *is an anaerobic gram-negative rod which is considered to be one of the major etiological agents of chronic periodontitis [1]. The local host response to aa includes the recruitment of neutrophils and the subsequent release of inflammatory mediators and cytokines, which appear to play an important role in the pathogenesis of periodontal disease. The mechanisms responsible for gingival tissue damage are poorly understood, and both immune-mediated reactions and direct cytopathic effects of bacteria may be involved. Based on a direct effect of bacteria in cell cultures, it has been suggested that apoptosis might play an important role in periodontitis. However, the nature of molecular mechanisms participating in this process remain unknown

Programmed cell death or apoptosis is a normal physiologic process that contributes to maintaining tissue homeostasis [3]. The process of apoptosis can be modulated by various stimuli, including hormones, cytokines, growth factors, bacterial or viral infections, and immune responses [4]. Recent studies demonstrate that apoptosis is essentially mediated by a family of cysteine proteases, called caspases, which can be grouped into initiator and effector caspases. Initiator caspases, such as caspase-8 or -9, exert regulatory roles by activating downstream effector caspases, such as caspase-3, -6, or -7, which cleave various cellular substrates [5]. Caspase-3 is considered an executor enzyme since it can be activated by several other active caspases, and because it has catalytic specificity for a relevant number of critical cellular substrates. One reliable indication of the induction of is detection of cells that express the active form of caspase-3 [6]. In fact, presence of cells that are positive for active caspase-3 is considered a hallmark of apoptosis activation.

Among other factors, the products of two genes that encode proteins p53 and *Bcl-2 *have been shown to play a fundamental regulatory role in apoptosis [7,8]. *Bcl-2 *is a member of a family of anti-apoptotic proteins that can prevent or reduce cell death induced by a variety of stimuli [9]. The intrinsic death pathway is initiated by the mitochondrial release of cytochrome *c*, a process that is inhibited by anti-apoptotic Bcl-2 proteins. Conversely p53 is the protein product of a tumor-suppressor gene, and expression of p53 can induce apoptosis. This protein is also implicated in the regulation of tissue dynamics, and is specifically thought to induce apoptosis in terminally differentiated cells, including inflammatory cells [10].

Various periodontal pathogens including *Porphyromonas gingivalis*, *Actinobacillus actinomycetemcomitans*, and *Eikenelle Corrodens *have been reported to induce cytotoxicty in a variety of cellular components of the periodontium. Based on a direct cytopathic effect of bacteria in cell cultures, it has been suggested that apoptosis might play an important role in periodontitis [11-13]. Moreover, lipopolysaccharides (LPS), a common component of the cell wall of gram-negative bacteria stimulate butyric acid-induced apoptosis in human peripheral blood mononuclear cells, and a toxin from aa induces apoptosis in B lymphocytes present in the periodontal tissue [11,14,15]. The induction of apoptosis in the host's cells provoked by certain pathogens, or their products, is a phenomenon involved in the pathogenesis of periodontal diseases. Bacterial phagocytosis or exposure to different bacterial components such as LPS, may delay apoptosis of the PMNs [16]. Berker et al demonstrated that neutrophil apoptosis provided a signal to monocytes, changing the phenotype of the monocyte resulting in the production of anti-inflammatory cytokines and suppression of proinflammatory cytokines in response to LPS [17].

All these data indicate that apoptotic mechanisms seem to play an important role in the pathogenesis of periodontal diseases. Despite the elucidation of apoptotic signaling cascades, it is almost completely unknown whether and to which extent caspases are activated in human gingival pathologies. The aim of this study was to compare quantities of immunohistochemically identified p53, *Bcl-2 *and caspase-3 in gingival tissue from patients with GAP and healthy subjects.

## Methods

### Selection of patients

The criterion for inclusion of GAP patients (3 males and 5 females; age range, 26–39 years; mean age, 34.12 ± 4.54 years) was generalized loss of proximal attachment, affecting at least 3 teeth other than the incisors and first molars. At least 5 or 6 teeth in each GAP patient had sites with PD ≥ 5 mm (mean PD: 4.4 ± 0.7 mm), and all selected patients showed extensive associated bone loss on radiographs At the time of examination, none of the GAP patients had been treated. The 10 control subjects (4 males and 6 females; age range, 14–32 years; mean age, 24.70 ± 7.13 years) had no history of periodontal disease.

None of the subjects had any known systemic disorder or had used antibiotics and anti-inflammatory medications in the last 3 months. Patient and control subjects with active infectious diseases such as hepatitis, HIV infection, and tuberculosis or chronically treated with medications (phenytoin, cyclosporin-A, or calcium channel blockers), as well as females, who were lactating or pregnant, were excluded. Probing depths in control group were <3 mm, and none of the controls showed loss of attachment, clinical evidence of inflammation, or bone loss on radiographs.

### Clinical examination and measurements

In each of the 18 total subjects, the quantity of microbial dental plaque present was determined using the Silness and Löe plaque index (PI) [18], and gingival status was assessed using the Löe and Silness gingival index (GI) [19]. Periodontal probing depths (PD) were measured to the nearest millimeter using periodontal probe. PI and GI were evaluated at 6 sites per tooth (mesio-vestibular, mid-vestibular, disto-vestibular, mesio-palatinal, mid-palatinal, and disto-palatinal). Clinical parameters were recorded for the entire dentition.

### Sampling of gingival tissue

Gingival tissue biopsies were obtained under local anesthesia from 8 patients diagnosed with GAP. The site with probing depth >5 mm were chosen for biopsy in GAP patients. In inflamed tissues, incisions were made 1 to 2 mm subgingivally. The specimens consisted of gingival epithelium and gingival connective tissue. The control specimens were obtained from 10 healthy controls with no periodontal disease during extraction of third molars Informed consent was obtained before the biopsy procedure, and one sample was collected from each subject.

### Tissue processing and immunohistochemistry

All biopsies were fixed in formalin and embedded in paraffin blocks. Several 4 mm-thick sections were obtained from each block and prepared with hematoxylin and eosin (H&E). Briefly, for immunohistochemical study, 3 mm-thick sections were deparaffinized and mounted on slides coated with poly-L-lysine. The sections were placed in citrate buffer (0.01 mol/L, pH 6), heated in a microwave oven for 15 minutes at maximum power (700 W), and then cooled at room temperature for 20 minutes. The sections were then incubated with primary antibodies for p53 (Ready-to-use Ab-5, NeoMarkers, Fremont, CA, USA), *Bcl-2 *(Ready-to-use Ab-1, NeoMarkers, Fremont, CA, USA), and caspase-3 (Ready-to-use Ab-5, NeoMarkers, Fremont, CA, USA) for 2 hours in a humidified chamber at room temperature. After washing in buffer, each slide was incubated with biotinylated goat anti-polyvalent for 15 minutes at room temperature, and thereafter in streptavidine peroxidase for another 15 minutes at room temperature. Slides were then developed for 12 minutes in diaminobenzidine (DAP, NeoMarkers, Fremont, CA, USA), followed by counterstaining with hematoxylin. Colon adenocarcinoma (for p53) and tonsil tissue (for *Bcl-2*) were used as positive controls for immunostaining.

Levels of expression of p53 and *Bcl-2 *on each slide were graded in a semiquantitative fashion using a scale of 0 to 3+: (0) = no staining; (1+) = stained cells comprising >10% of the inflammatory infiltrate (2+) = stained cells comprising 30% of the inflammatory infiltrate, and (3+) = stained cells comprising >30% of the inflammatory infiltrate. Expression of caspase-3 was graded in a semiquantitative manner using a two-tier scale: (0) = staining cells <30% of the inflammatory infiltrate or (1+) = staining >30% of the inflammatory infiltrate.

### Statistical analysis

Data for the clinical parameters were expressed as mean ± standard deviation. Differences in clinical parameters and histological findings between the GAP and control groups were analyzed using the Student's *t*-test. A statistical software package (SPSS), *Version 10.0 for Windows, SPSS Inc., Chicago, IL*.) was used and changes were considered significant at the p < 0.05 levels.

## Results

The data for age and periodontal status in both groups are presented in Table 1. Eight patients (mean age 34.12 ± 4.54) with GAP and ten healthy individuals (mean age 24.7 ± 7.13) were selected for this study. There were significant differences between the groups with respect to age and PD, but not GI or PI (*P *< 0.05).

**Table 1:** Mean clinical findings in GAP patients and controls.

	**GAP Group (N = 8)**	**Control Group (N = 10)**
Age (yrs)	34.12 ± 4.54 *	24.7 ± 7.13
Probing depth (mm)	4.44 ± 0.75 *	2.22 ± 0.36
Gingival index	1.01 ± 0.21	1.10 ± 0.32
Plaque index	1.52 ± 0.54	1.10 ± 0.29

* Significant difference (*P *< 0.05)

**Figure 1:**
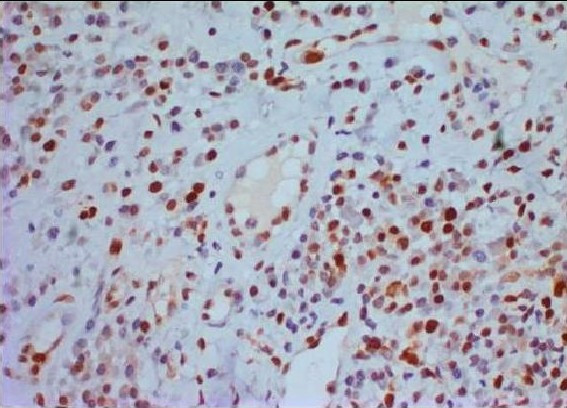
Significant nuclear staining for p53 was observed in the mononuclear inflammatory cells in the GAP group specimens. (×200 *p53 *immunostaining).

**Figure 2:**
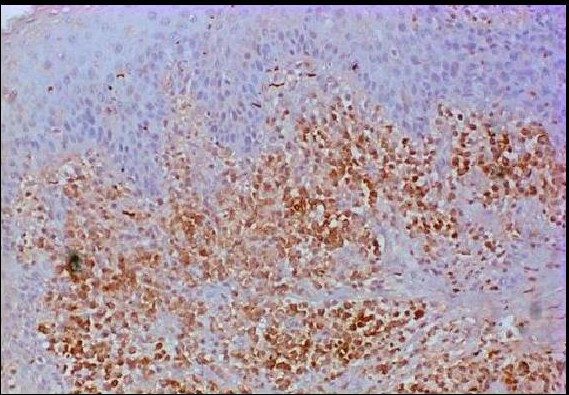
The diffuse mononuclear inflammatory cell infiltrate beneath the epithelium in a GAP specimen shows significant *Bcl-2 *staining. (×100 *Bcl-2 *immunostaining).

**Figure 3:**
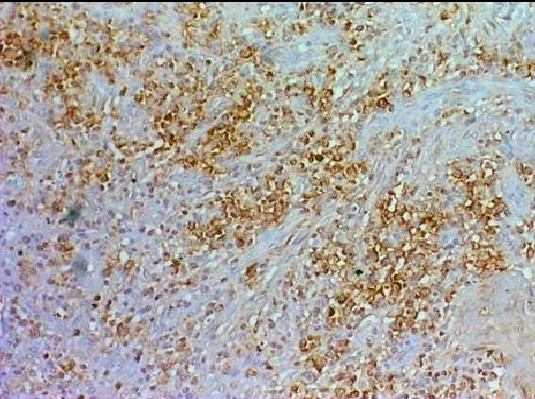
Diffuse significant caspase-3 staining was observed in the inflammatory cells in the GAP specimens. (×200 Caspase-3 immunostaining).

Immunohistochemical findings are shown in Table 2. There were no differences between the groups with respect to grades of caspase-3 and p53 expression (Figures 1 and 2, respectively) (*P *> 0.05). However, the frequency of grade 3+ expression of *Bcl-2 *was significantly higher in the GAP group than that of the control group (Figure 3) (*P *< 0.05).

**Table 2:** The distribution of stained cells in GAP patients and the controls.

	**GAP Group**		**Control Group**	
	
	**N**	**%**	**N**	**%**
Cas-3 grade 0	4	50	6	60
Cas-3 grade 1	4	50	4	40

p53 grade 1	-	-	4	40
p53 grade 2	5	62.5	2	20
p53 grade 3	3	37.5	4	40

Bcl-2 grade 1	-	-	6	60
Bcl-2 grade 2	2	25	4	40
Bcl-2 grade 3	6	75 *	-	-

* Significant difference (*P *< 0.05)

## Discussion

While GAP affects a minority of periodontal patients, it is highly significant due to severe tissue destruction. Although the presence of bacterial pathogens is necessary for the initiation of periodontal diseases, complex inflammatory and immune response also play a critical role in the progression of disease [20,21].

Apoptosis, or programmed cell death, plays a critical role in the regulation of inflammation and the host immune response. During this process, a series of coordinated morphological and biochemical events is induced in the affected cell, resulting in its death and subsequent removal by scavenger phagocytes [3]. Gamonal and coworkers studied gingival tissues from patients with chronic periodontitis and from healthy controls [22]. They examined sections of gingival tissues by electron microscopy and performed immunohistochemical analysis to detect DNA fragmentation-positive cells and active caspase 3, Fas/FasL, Bcl-2 and p53-positive cells. Positive staining for active caspase-3, *Fas*, *Fas*L and p53 in the inflammatory infiltrates was only observed in the inflammatory infiltrate from the chronic periodontitis biopsies, whereas *Bcl-2*-positive cells was present in the tissues from both groups. In this study, they demonstrated the presence of apoptotic cells by electron microscopy in the deep area of biopsies from sites with probing depth ≥ 5 mm and attachment with ≥ 3 mm. These results showed that apoptotic cells were detected in the gingival tissue adjacent to >6 mm sulci. This study strongly supported the relevance of the apoptotic mechanisms in the selection of immunocompotent cell population in periodontal tissues.

Our study compared clinical findings and expression of p53, *Bcl-2 *and caspase-3 in patients with GAP versus healthy controls. We found no significant differences between these groups with respect to overall frequency of caspase-3 expression or frequencies of different grades of expression of p53. However, staining for *Bcl-2 *was more frequent in the GAP specimens than in the controls.

Certain activators of apoptosis require the presence of a functional p53 protein. P53 is a tumor suppressor protein which, when active, induces genes related to cell cycle regulation, DNA repair mechanisms, and the induction of apoptosis [23]. Although p53 is present in normal tissues and cells, its short half-life make its expression almost undetectable in healthy normal tissues [24]. Upon activation, p53 stabilized so that its expression can be detected with anti-p53 antibodies using standard immunohistochemical techniques. This explain our finding that the staining cell of p53 was similar between in both groups. Thus, our data suggests that the exact mechanism of p53-dependent apoptosis remains to be identified.

Apoptosis may therefore be an important phenomenon in the regulation of the inflammatory response against chronic bacterial accumulation, affecting both the increase in cellularity and extent of the inflammatory infiltration [25]. The process of apoptosis involves activation of a series of cysteine-type proteases that are named caspases because of their catabolic properties [10]. These enzymes are synthesized as inactive proenzymes, which are then processed in cells that undergo apoptosis. Caspase-3 is a key protease that is activated during apoptosis. Detection of active caspase-3-positive inflammatory cells is evidence that apoptotic mechanisms are probably underway. However, the small numbers of positive cells for these apoptosis markers suffer a significant loss of affinity to tissue, suggesting that the tissue destruction taking place in this condition may occur after a long time of evolution. Although we aimed to evaluate the reduction of apoptosis in the GAP group, we could not demonstrate any significant difference between the positive staining cells of caspase-3 in both groups. An increase in sample size may result in statistical significance in further studies.

Mamalian *Bcl-2 *protein family of apoptosis-associated proteins consists of members that inhibit apoptosis (Bcl-2, Bcl-xl) and others that induced apoptosis (Bax, Bak, etc.) [26]. Aberrant apoptosis regulation is considered to contribute to autoimmune disorders such as systemic erythematosus such as rheumatoid arthritis, viral diseases including AIDS and bacterial infection [27-29]. Therefore, abnormalities in the regulation of cell homeostasis may contribute to a number of different pathogenic processes. As noted, these anti-apoptotic proteins can prevent or limit cell death induced by different stimuli [30,31]. Recent studies show that the bacterial products isolated from different strains Porphyromonas gingivalis delay neutrophil apoptosis in a dose-dependent fashion [32]. Certain cytokines increase the lifespan of the neutrophils by inhibiting their apoptotic cell death in vitro and this prolonged neutrophil survival has been associated with an enhanced inflammatory response [33,34]. Gamanol et al showed a positive correlation between the delay in the apoptotic process of neutrophils in periodontal tissues with an increase in the levels of tumor necrosis factor-alpha (TNF-α), granulocyte monocyte-colony stimulating factor (GM-CSF) and low expression of Bax, thus suggesting a possible role of these soluble mediators in the pathogenesis of periodontitis [35]. The higher frequency of *Bcl-2 *positivity in our GAP group indicates that delayed apoptosis can lead to inflammatory cells staying locally in periodontal tissues longer and thus secreting excessive cytokines, finally to progressive periodontal destruction.

Our data indicate that host-mediated mechanisms are involved in periodontitis-associated tissue damage. Moreover, the finding that tissue injury is associated with elevated Bcl-2 activation could open up new diagnostic possibilities and therapeutic strategies to prevent tissue destruction in periodontal disease.

## Competing interests

The author(s) declare that they have no competing interests.

## Authors' contributions

SB concieved and coordinated the study and participated in the collection of sample and data; and writing the manuscript. HU participated in the collection of samples and writing the manuscript. BHÖ carried out tissue processing and immunohistochemistry. SB, BHÖ and ÖEB analyzed the data. ÖEB participated in the design of the study and performed statistical analysis. All authors read and approved the final manuscript.
